# Supplementary material for: Retrotransposons in Werner syndrome-derived macrophages trigger type I interferon-dependent inflammation in an atherosclerosis model
Source: Nat Commun. 2024 Jun 10;15:4772. doi: 10.1038/s41467-024-48663-w (PMC11164933; doi:10.1038/s41467-024-48663-w)
Supplement: Supplementary file 1 — Supplementary Information [file 41467_2024_48663_MOESM1_ESM.pdf]

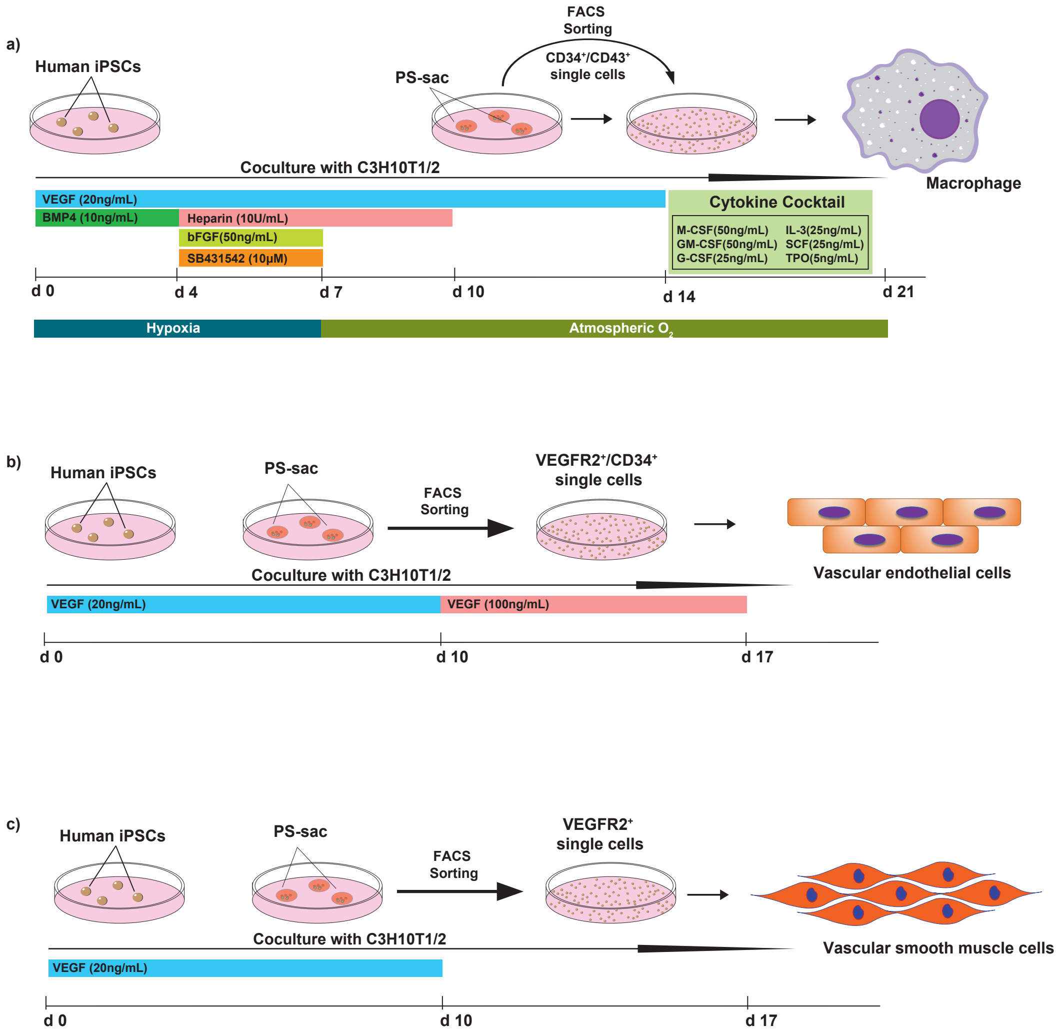

Supplementary Figure 1. Scheme of induction of Mφs and vascular cells from human iPSCs. Schematic diagrams of *in vitro* protocols for differentiation of human iPSCs into **a)** Mφs, **b)** VECs, and **c)** VSMCs. C3H10T1/2, mouse mesenchymal stromal cells; VEGF, vascular endothelial growth factor; BMP4, bone morphogenetic protein 4; bFGF, basic fibroblast growth factor; GM-CSF, granulocyte-macrophage colony-stimulating factor; M-CSF, macrophage colony-stimulating factor; G-CSF, granulocyte-colony stimulating factor; IL-3, interleukin 3; SCF, stem cell factor TPO, thrombopoietin; VEGFR2, vascular endothelial growth factor receptor 2.

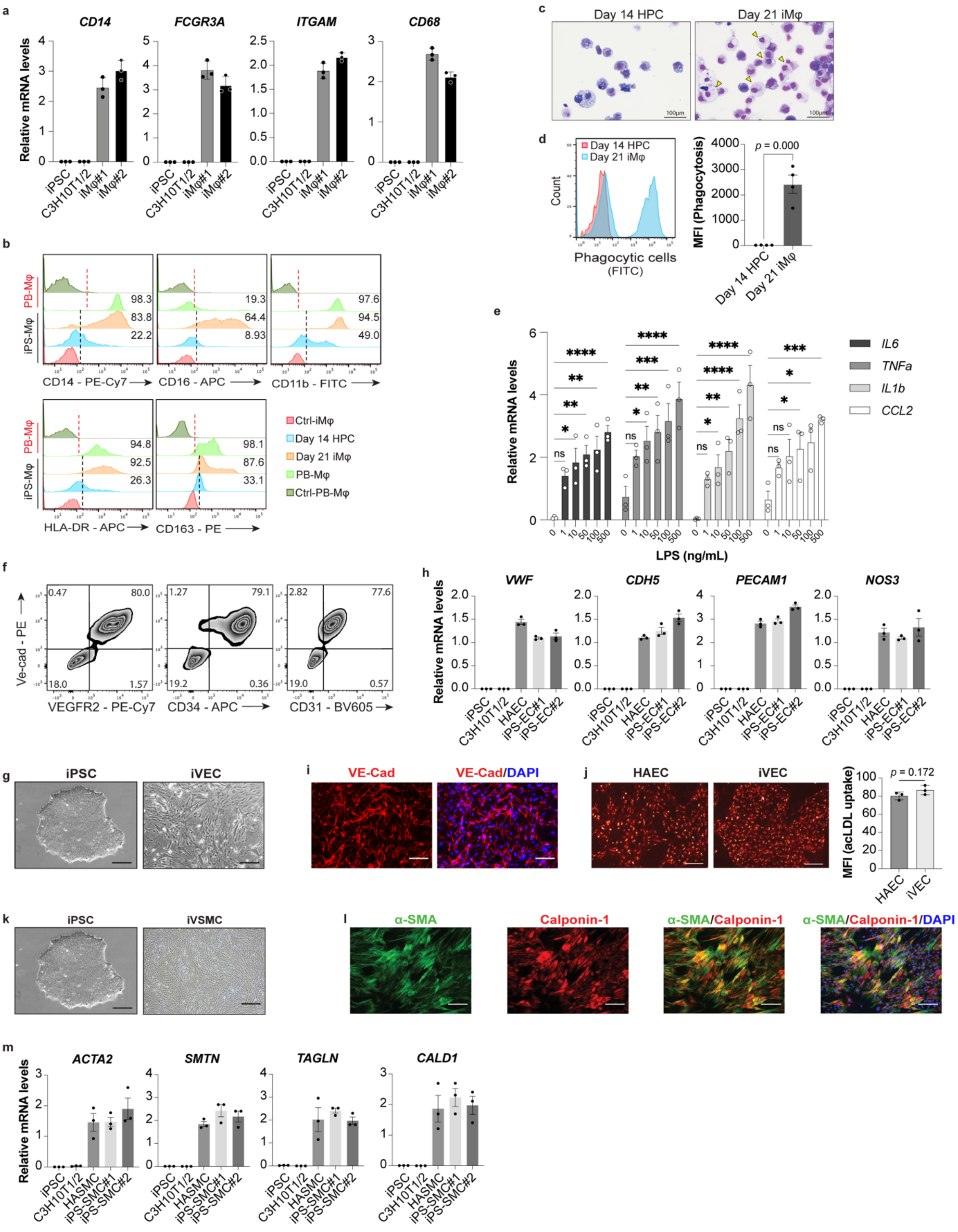

Supplementary Figure 2. Differentiation and characterization of human iPSC-derived Mφs and vascular cells. a) Representative bar graphs showing mRNA levels of hematopoietic and Mφ-specific genes normalized by *GAPDH* mRNA (n = 3). **b)** Flow cytometric histograms of the expression of cell surface markers on iPS- and PB-derived Mφs. iPS-derived Mφs were stained with appropriate

antibodies on days 14 and 21 of differentiation. Unstained cells were used as negative controls for each cell type. **c)** Giemsa-stained images of day 14 HPCs (left) and day 21 mature iMφs (right). **d)** Representative flow cytometry plots for phagocytosis assay on days 14 and 21 of differentiation (left) and MFI of phagocytosis (right) (n = 4). An unpaired *t*-test was performed to calculate the p-value. **e)** mRNA levels of pro-inflammatory cytokines after dose-dependent treatment of lipopolysaccharide normalized by *GAPDH* mRNA on day 21 of differentiation (n = 3). **f)** Flow cytometric analysis of different VEC cell surface markers on day 17 of differentiation. **g)** Phase-contrast microscopic images of iPSCs (left) and mature iVECs (right) on day 17 of differentiation. The scale bar is 500μm. **h)** mRNA expression levels of VEC maturation genes normalized by *GAPDH* mRNA on day 17 of differentiation (n = 3). **i)** Immunocytochemistry of VE-cad expression (left) and VE-cad with DAPI (right). The scale bar is 100μm. **j)** Acetylated low-density lipoprotein (acLDL) uptake between primary human aortic endothelial cells (HAECs; far left) and iVECs (middle) and MFI of acLDL (right) (n = 3). An unpaired *t*-test was performed to calculate the p-value. The scale bar is 500μm. **k)** Phase-contrast microscopic image of iPSCs (left) and mature iVSMCs on day 17 of differentiation (right). The scale bar is 500μm. **l)** Immunocytochemistry of α-SMA (far left), calponin-1 (left), α-SMA with calponin-1 (right), and α-SMA with calponin-1 with DAPI (far right). The scale bar is 100μm. **m)** mRNA expression levels of VSMC maturation genes normalized by *GAPDH* mRNA on day 17 of differentiation (n = 3). Data are shown as the mean ± SEM of biologically independent samples. *p < 0.05, **p < 0.01, ***p < 0.001, ****p < 0.0001. ns, not significant. Source data are provided as a Source Data file.

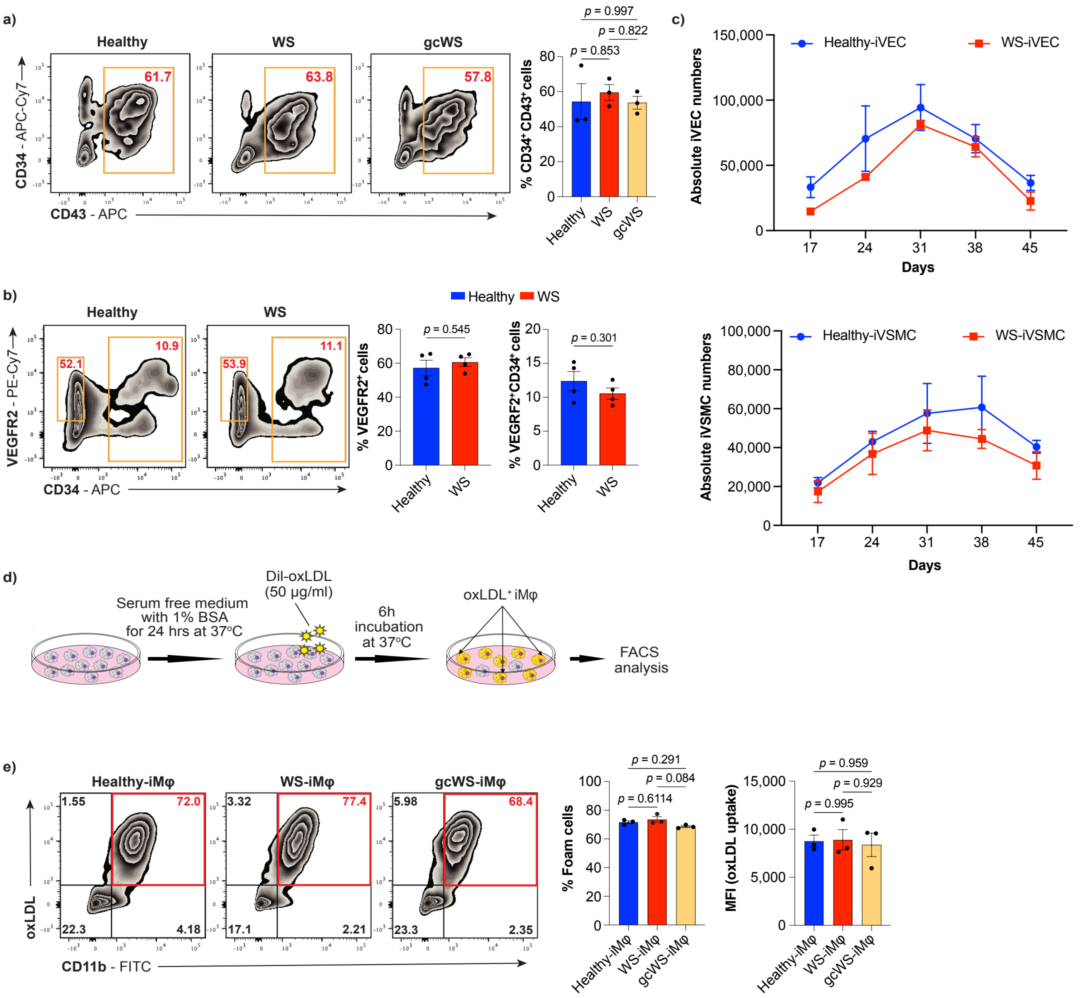

Supplementary Figure 3. Comparisons among healthy-, WS-, and gcWS-iMφs and iPSC-derived vascular cells. a) Flow cytometric analysis of cell surface markers of HPCs (left) and representative bar graph of $CD34^+$ $CD43^+$ cells (right) induced from healthy-, WS- and gcWS-iPSCs on day 14 of differentiation (n = 3). One-way ANOVA with Tukey's multiple comparisons was performed to calculate the *p* values. **b)** Flow cytometric analysis of cell surface markers of VPCs (left) and representative bar graph of $VEGFR2^+$ $CD34^-$ cells (middle) and $CD34^+$ cells (right) induced from healthy- and WS-iPSCs on day 10 of differentiation. (n = 4). An unpaired *t*-test was performed to calculate the p-value. **c)** Absolute numbers of healthy- and WS-iVECs (top) and healthy- and WS-iVSMCs (bottom) on day 17 of differentiation. **d)** Schematic diagram of oxLDL treatment in iMφs on day 21 of differentiation. **e)** Flow cytometric analysis of oxLDL uptake by healthy-, WS-, and gcWS-iMφs (left) and calculated percent foam cell formation (middle) and MFI of oxLDL uptake (right) (n = 3). One-way ANOVA with Tukey's multiple comparisons was performed to calculate the *p* values.

Percent foam cell formation was calculated from the oxLDL^{+} CD11b^{+} cell population. Data are shown as the mean ± SEM. (n = 4) two biologically independent samples over two independent experiments. (n = 3) represents biologically independent samples. Source data are provided as a Source Data file.

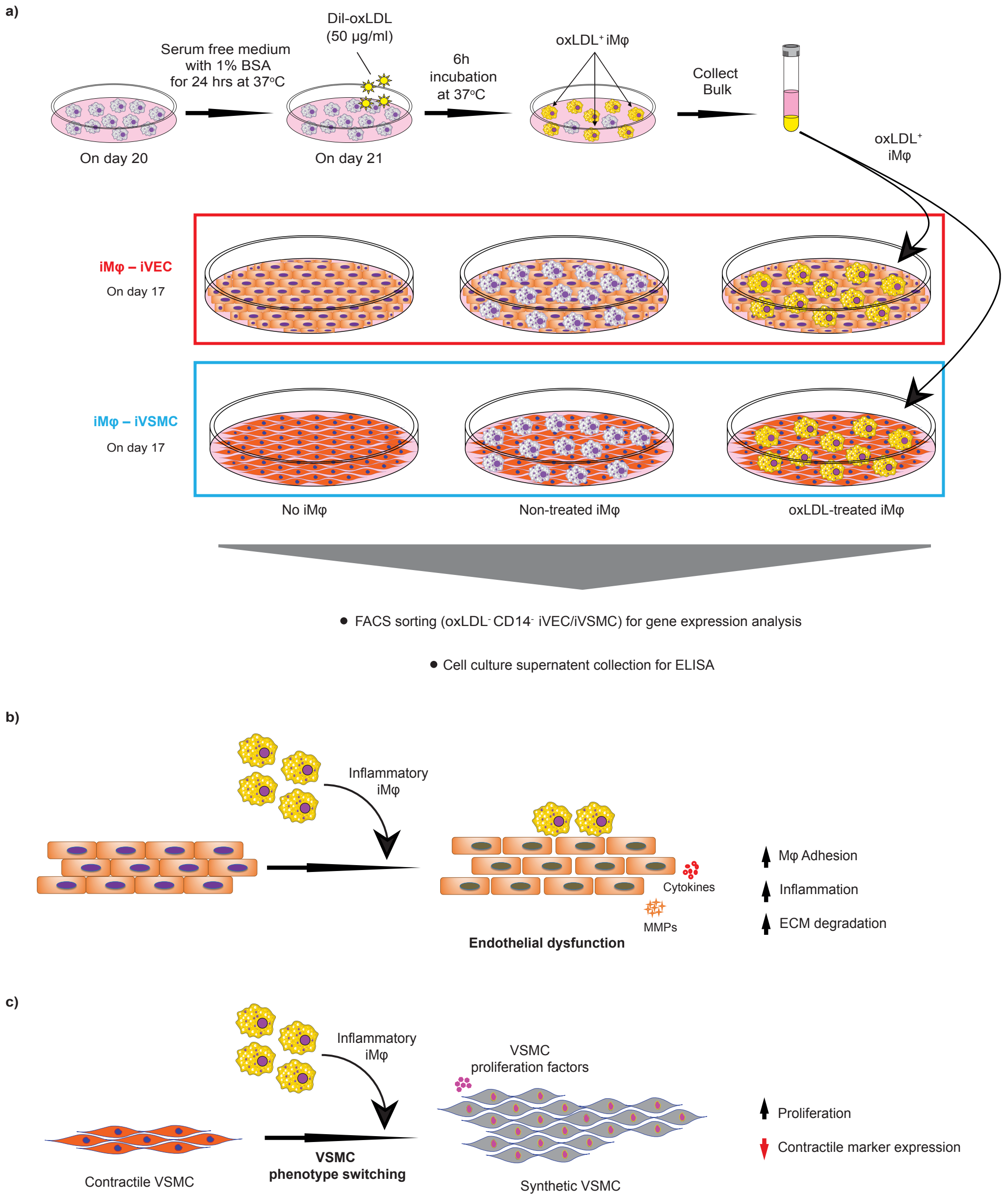

Supplementary Figure 4. *In vitro* iMφ and iPSC-derived vascular cell co-culture system. a) Schematic diagrams of *in vitro* 2D co-culture of iMφs with iPSC-induced vascular cells. **b)** Schematic diagram of endothelial dysfunction of WS-iVECs after co-culture with iMφs. **c)** Schematic diagram of VSMC phenotype switching of WS-iVSMCs after co-culture with inflammatory iMφs.

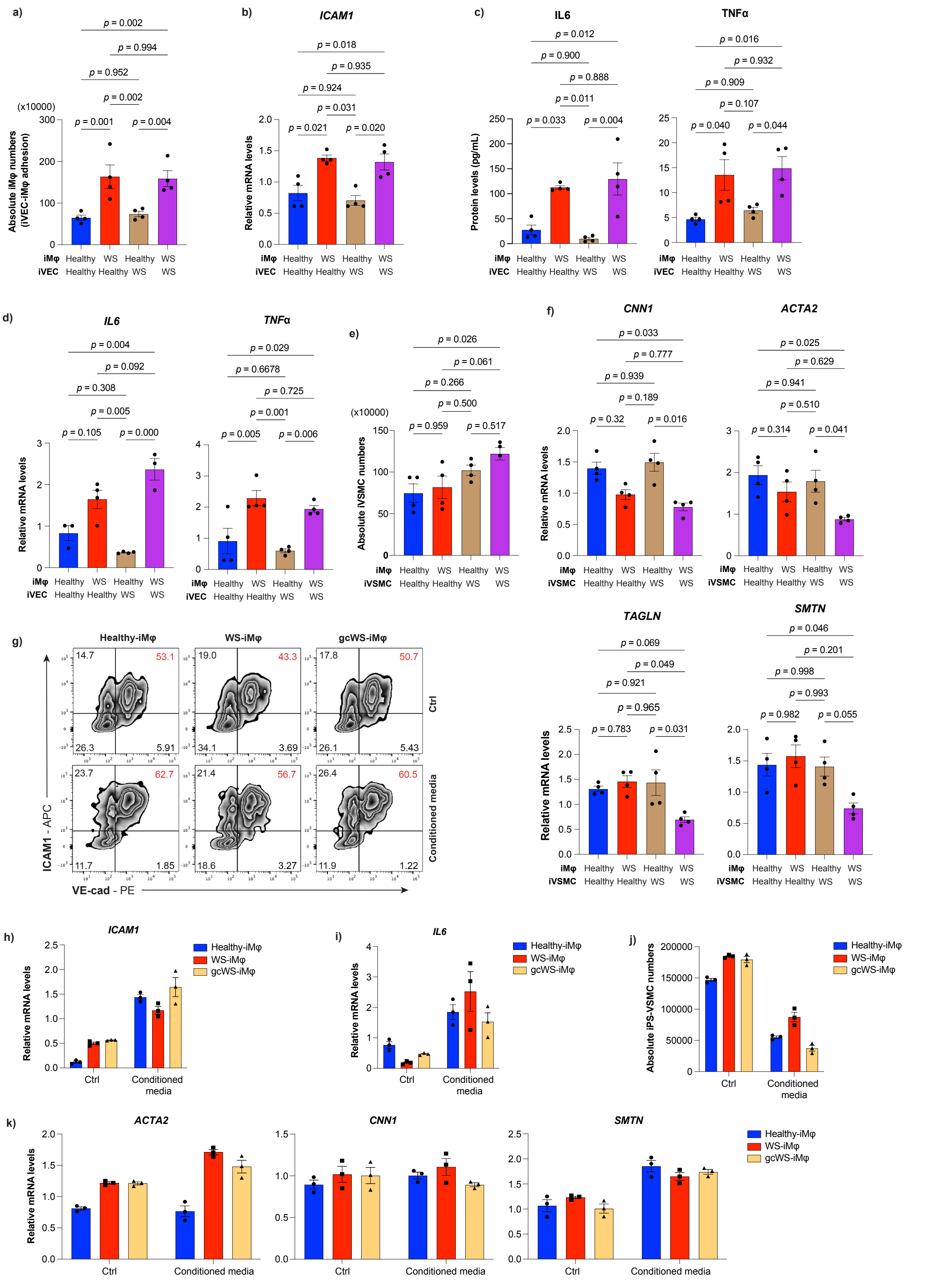

Supplementary Figure 5. Cross-co-culture showed that WS-iMφs were necessary for inducing phenotypic changes in vascular cells. a) Absolute numbers of CD14^{+} adherent iMφs on iVECs after

co-culture with oxLDL-treated iMφs (n = 4). **b)** mRNA levels of *ICAM1* normalized by *GAPDH* mRNA in iVECs after co-culture with oxLDL-treated Mφs (n = 4). **c)** IL6 and TNFα protein levels quantified by ELISA after co-culture with oxLDL-treated Mφs (n = 4). **d)** *IL6* and *TNFα* mRNA levels normalized by *GAPDH* mRNA in iVECs after co-culture with oxLDL-treated Mφs (n = 4). **e)** Absolute numbers of iVSMCs after co-culture with oxLDL-treated Mφs (n = 4). **f)** mRNA levels of VSMC contractile markers (*CNN1* (n = 4), *ACTA2* (n = 4), *TAGLN* (n = 4), and *SMTN* (n = 5)) normalized by *GAPDH* mRNA after co-culture with oxLDL-treated Mφs. **g)** Flow cytometric analysis of cell surface marker (ICAM-1 and VE-cad) expression on iVECs in control and CM groups. **h)** *ICAM-1* mRNA levels normalized by *GAPDH* mRNA in control and CM groups (n = 3). **i)** *IL6* mRNA levels normalized by *GAPDH* mRNA in the control and CM group (n = 3). **j)** Absolute numbers of iVSMCs in control and CM groups (n = 3). **k)** mRNA levels of VSMC contractile markers normalized by *GAPDH* mRNA in control and CM groups (n = 3). Two-way ANOVA with Tukey's multiple comparisons was performed to calculate the *p* values. Data are shown as the mean ± SEM. (n = 4) represents two biologically independent samples over two independent experiments, and (n = 3) represents biologically independent samples. Source data are provided as a Source Data file.

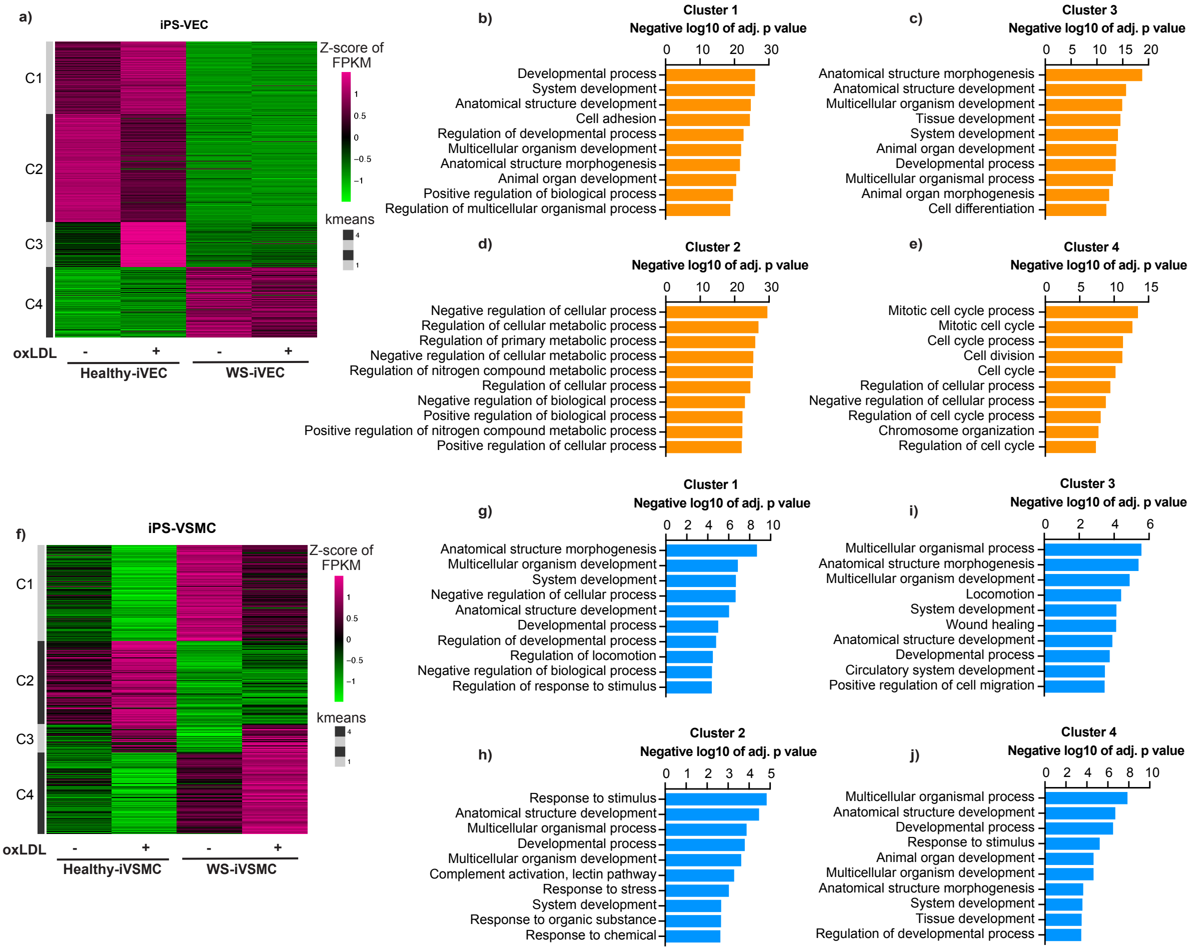

Supplementary Figure 6. RNA-seq analysis of iPS-derived vascular cells. a) Heatmap of DEGs in healthy- and WS-iVECs before and after oxLDL treatment. DEGs were obtained from Cuffdiff and grouped into four clusters by K-means clustering, with each column representing a sample group and each row representing an individual gene (n = 3 biologically independent samples). Cluster-wise pathway enrichment analysis of the top 10 pathways in each cluster in iVECs **(b-e)**. **f)** Heatmap of DEGs in healthy- and WS-iVSMCs before and after oxLDL treatment. DEGs were obtained from Cuffdiff and grouped into four clusters by K-means clustering, with each column representing a sample group and each row representing an individual gene (n = 3 biologically independent samples). Cluster-wise pathway enrichment analysis of the top 10 pathways in each cluster in iVSMCs **(g-j)**. Source data are provided as a Source Data file.

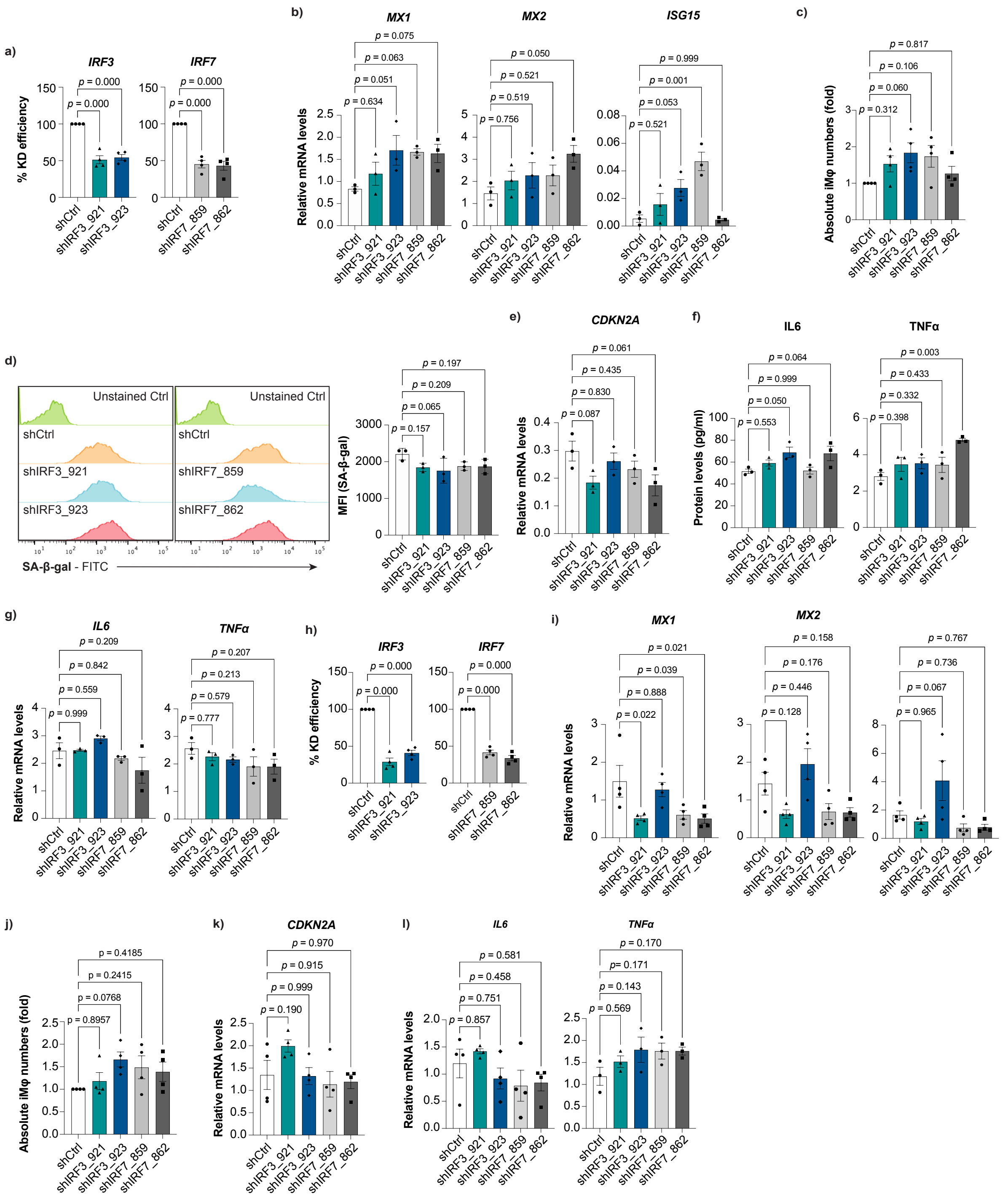

Supplementary Figure 7. Effect of type I IFN signal knockdown on healthy- and gcWS-iMφs. a) Percent knockdown (KD) efficiency by shIRF3 and shIRF7 in healthy-iMφs (n = 4). **b)** mRNA levels of type I IFN signature genes normalized by *GAPDH* mRNA after lentiviral transduction of shIRF3 and shIRF7 in healthy-iMφs (n = 3). **c)** Fold change in absolute numbers of healthy-iMφs after lentiviral transduction of shIRF3 and shIRF7 (n = 4). **d)** Representative flow cytometric plots of SA-β-gal staining

after lentiviral transduction of shIRF3 (left) and shIRF7 (middle) and MFI of SA-β-gal (right) (n = 3). **e)** *CDKN2A* mRNA levels normalized by *GAPDH* mRNA in healthy-iMφs (n = 3). **f)** Pro-inflammatory cytokine levels, determined by ELISA, after lentiviral transduction of shIRF3 and shIRF7 in healthy-iMφs (n = 3). **g)** *IL6* and *TNFα* mRNA levels normalized by *GAPDH* mRNA after lentiviral transduction of shIRF3 and shIRF7 in healthy-iMφs (n = 3). **h)** Percent KD efficiency by shIRF3 and shIRF7 in gcWS-iMφs (n = 4). **i)** mRNA levels of type I IFN signature genes normalized by *GAPDH* mRNA after lentiviral transduction of shIRF3 and shIRF7 in gcWS-iMφs (n = 4). **j)** Fold change in absolute numbers of gcWS-iMφs after lentiviral transduction of shIRF3 and shIRF7 (n = 4). **k)** *CDKN2A* mRNA levels normalized by *GAPDH* mRNA in gcWS-iMφs (n = 4). **l)** *IL6* and *TNFα* mRNA levels normalized by *GAPDH* mRNA after lentiviral transduction of shIRF3 and shIRF7 in gcWS-iMφs (n = 4). Data are shown as the mean ± SEM. (n = 4) represents two biologically independent samples over two independent experiments, and (n = 3) represents biologically independent samples. One-way ANOVA with Dunnett's multiple comparisons was performed to calculate the *p* values. Source data are provided as a Source Data file.

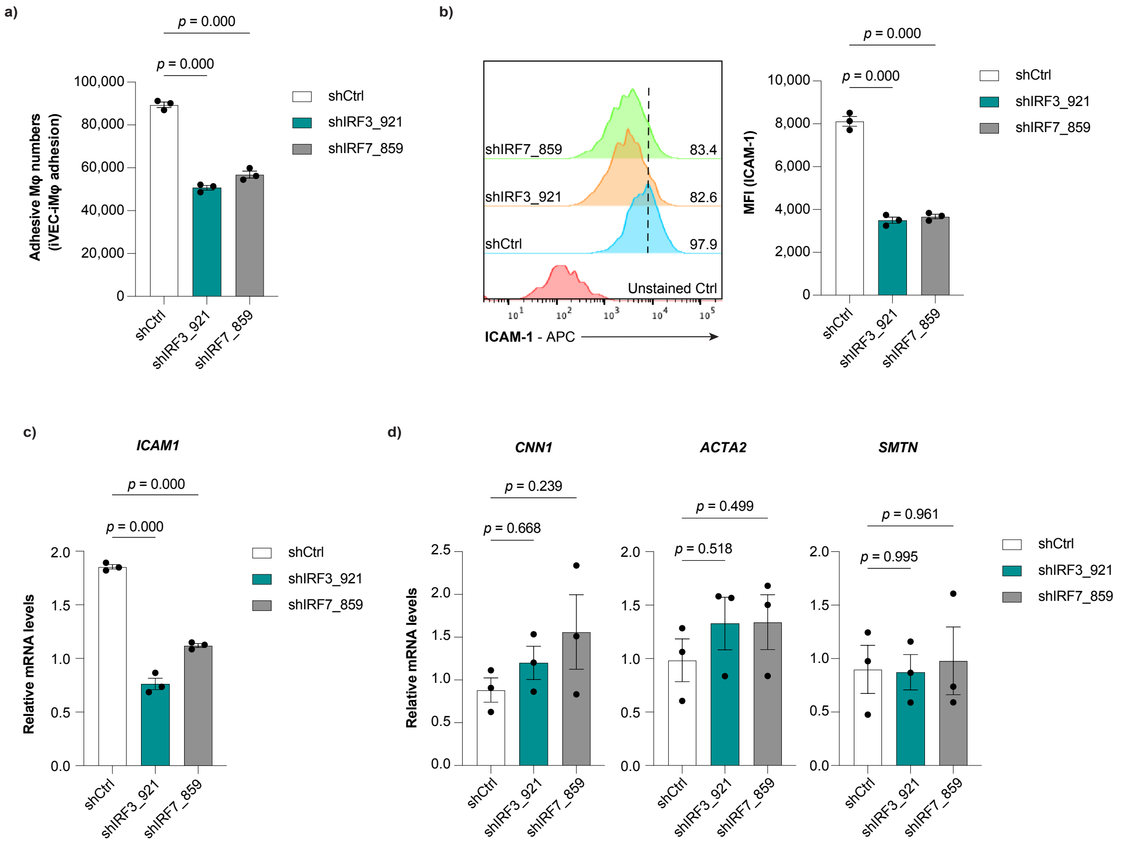

Supplementary Figure 8. Silencing type I IFN signaling ameliorates vascular health in WS-iPS-derived cells. a) Absolute numbers of CD14^{+} adherent iMφs on iVECs after co-culture with shIRF3 and shIRF7 lentiviral-transduced oxLDL-treated WS-iMφs (n = 3). **b)** Representative FACS histograms (left) and MFI (right) of cell surface ICAM-1 expression on iVECs. The dotted line represents the shCtrl histogram peak (n = 3). **c)** *ICAM-1* mRNA levels normalized by *GAPDH* mRNA (n = 3). **d)** mRNA levels of VSMC contractile markers normalized by *GAPDH* mRNA in iVSMCs after co-culture with shIRF3 and shIRF7 lentiviral-transduced oxLDL-treated WS-iMφs (n = 3). Data are shown as the mean ± SEM of biologically independent samples. One-way ANOVA with Dunnett's multiple comparisons was performed to calculate the *p* values. Source data are provided as a Source Data file.

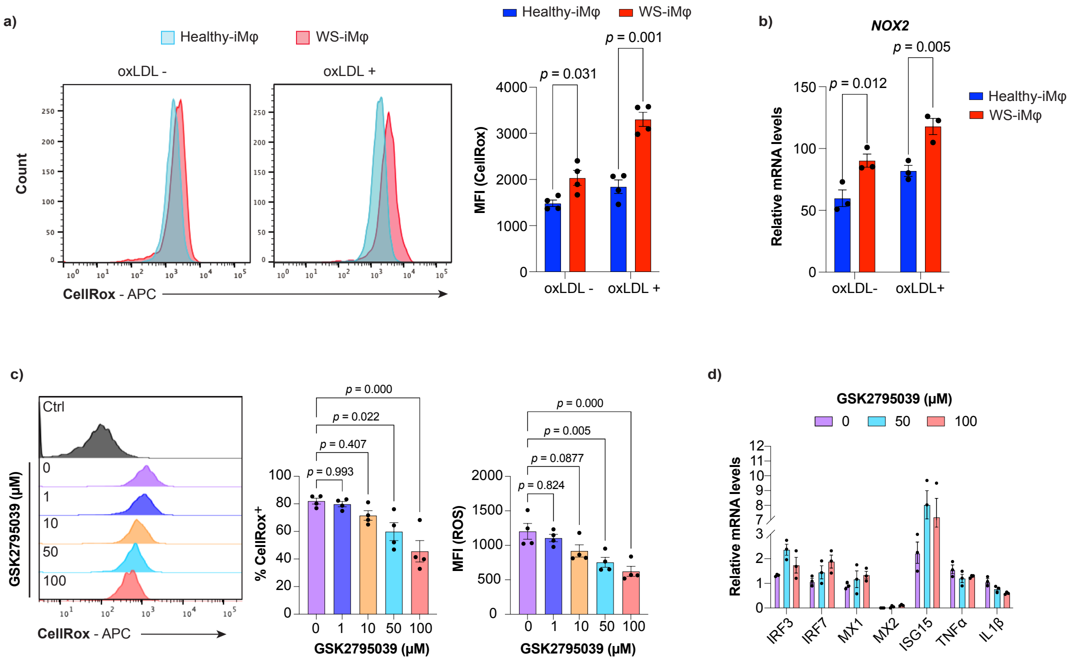

Supplementary Figure 9. Unchanged type I IFN signaling upon *NOX2*-dependent inhibition of ROS generation in WS-iMφs. a) Representative flow cytometric plots showing ROS accumulation (left) and MFI of ROS accumulation (right) in healthy- and WS-iMφs before and after oxLDL treatment (n = 4). *t*-tests with correction for multiple comparisons using the Bonferroni-Dunn method were performed to calculate the *p* values. **b)** *NOX2* mRNA levels normalized by *GAPDH* mRNA (n = 3). *t*-tests with correction for multiple comparisons using the Bonferroni-Dunn method were performed to calculate the *p* values. **c)** Representative flow cytometric plots showing ROS accumulation (left), percent of ROS accumulated cells (middle), and MFI of ROS accumulation (right) in WS-iMφs before and after GSK2795039 treatment (n = 3). One-way ANOVA with Dunnett's multiple comparisons was performed to calculate the *p* values. **d)** mRNA levels of type I IFN signature and pro-inflammatory cytokine genes normalized by *GAPDH* mRNA. Data are shown as the mean ± SEM of biologically independent samples. Source data are provided as a Source Data file.

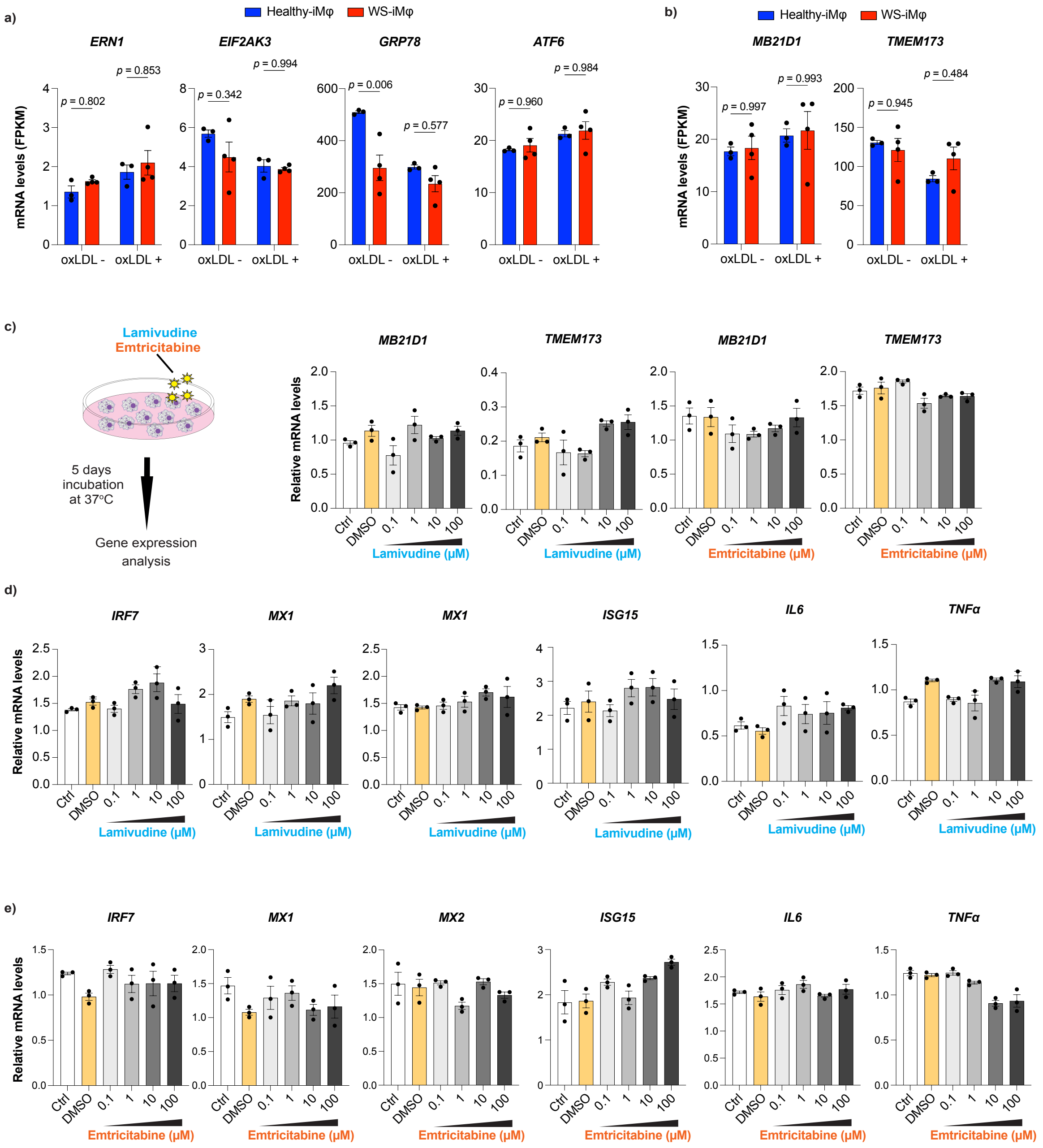

Supplementary Figure 10. Unchanged type I IFN signals upon NRTI treatment of WS-iMφs. a) FPKM of ER-stress-related genes in healthy- (n = 3) and WS-iMφs (n = 4) before and after oxLDL treatment. **b)** FPKM of *MB21D1* and *TMEM173* genes in healthy- (n = 3) and WS-iMφs (n = 4) before and after oxLDL treatment. **c)** Schematic diagram of nucleoside/nucleotide reverse transcriptase inhibitor (NRTI) treatment of WS-iMφs (left) and *MB21D1* and *TMEM173* mRNA levels in untreated and NRTI-treated WS-iMφs normalized by *GAPDH* mRNA. mRNA levels of type I IFN signature genes in untreated and lamivudine-treated **(d)** emtricitabine-treated **(e)** WS-iMφs normalized by

GAPDH mRNA. Two-way ANOVA with Tukey's multiple comparisons was performed to calculate the *p* values. Data are shown as the mean ± SEM of biologically independent samples. Source data are provided as a Source Data file. NRTI, Nucleoside Reverse Transcriptase Inhibitor.

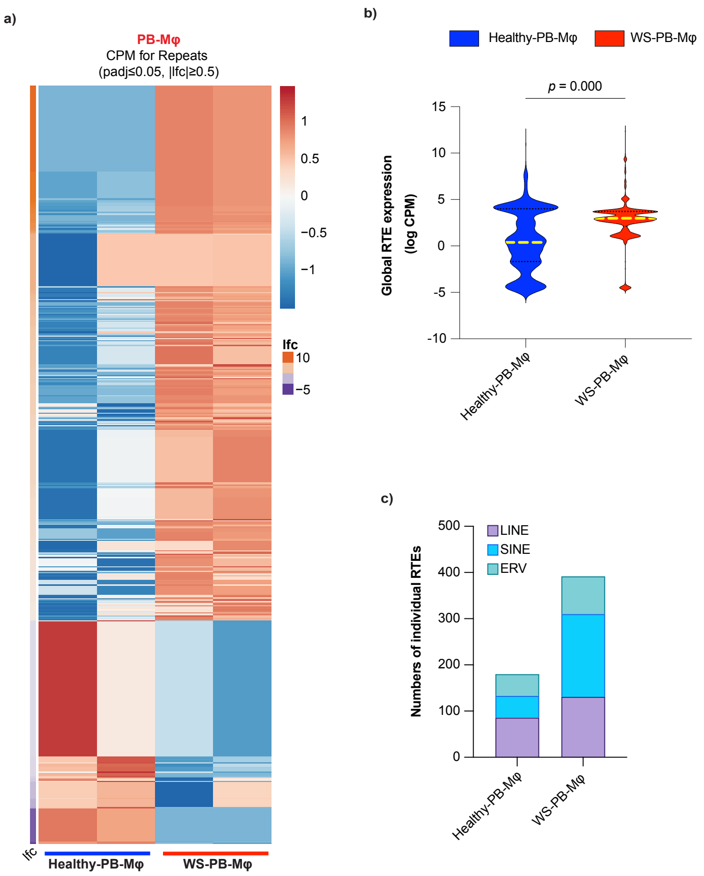

Supplementary Figure 11. Expression of RTEs in human peripheral blood-derived cells. a) Heatmaps showing differential expression of RTEs obtained using limma-voom in healthy aged-PB-Mφs (n = 2) and WS-PB-Mφs (n = 2), with each column representing a sample group and each row representing an individual RTE (adjusted p ≤ 0.05, lfc ≥ 0.5). Moderated paired *t*-tests were performed using limma, and the *p* values were corrected for multiple comparisons using Benjamini Hochberg's method. **b)** Levels of global RTE expression in healthy aged-PB-Mφs and WS-PB-Mφs (adjusted p ≤ 0.05, lfc ≥ 0.5). Data are presented as logCPM values of biologically independent samples. The yellow line on the violin plot represents the median global RTE expression. A two-tailed unpaired *t*-test was performed to calculate the *p* value **c)** Numbers of individual RTEs at sub-families (LINE, SINE, and ERV) levels in healthy aged-PB-Mφs and WS-PB-Mφs. Source data are provided as a Source Data file.

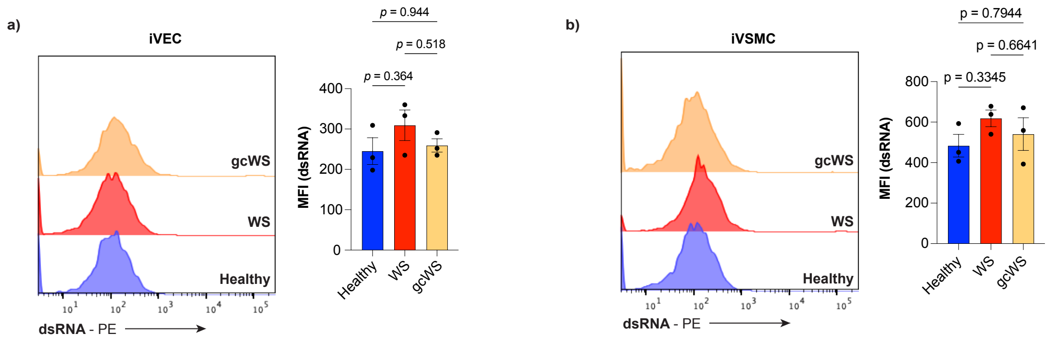

Supplementary Figure 12. Accumulation of dsRNA in iPS-derived vascular cells. a) Representative FACS plot of dsRNA accumulation (left) and MFI of dsRNA accumulation (right) in healthy-, WS-, and gcWS-iVECs (n = 3). **b)** Representative FACS plot of dsRNA accumulation (left) and MFI of dsRNA accumulation (right) in healthy-, WS-, and gcWS-iVSMCs (n = 3). One-way ANOVA with Tukey's multiple comparisons was performed to calculate the *p* values. Data are shown as the mean ± SEM of biologically independent samples. Source data are provided as a Source Data file.
