# Supplementary material for: Retrotransposons in Werner syndrome-derived macrophages trigger type I interferon-dependent inflammation in an atherosclerosis model
Source: Nat Commun. 2024 Jun 10;15:4772. doi: 10.1038/s41467-024-48663-w (PMC11164933; doi:10.1038/s41467-024-48663-w)
Supplement: Supplementary file 3 — Description of additional supplementary files [file 41467_2024_48663_MOESM3_ESM.pdf]

<u>Description of Additional Supplementary Files</u>

Supplementary Data 1. Results of GSEA analysis. Each tab contains a different pair-wise gene set enrichment analysis (GSEA). Normalized enrichment score (NES) and false direct recovery (FDR) qvalues were obtained from GSEA software.

Supplementary Data 2. Oligonucleotide sequences for RT-qPCR. All the oligonucleotide primers used in this study were listed.
