# Supplementary material for: Retrotransposons in Werner syndrome-derived macrophages trigger type I interferon-dependent inflammation in an atherosclerosis model
Source: Nat Commun. 2024 Jun 10;15:4772. doi: 10.1038/s41467-024-48663-w (PMC11164933; doi:10.1038/s41467-024-48663-w)
Supplement: Supplementary file 6 — Reporting Summary [file 41467_2024_48663_MOESM6_ESM.pdf]

nature portfolio

Corresponding author(s): Koutaro Yokote
Koji Eto
Naoya Takayama

Last updated by author(s): Apr 2, 2024

Reporting Summary

Nature Portfolio wishes to improve the reproducibility of the work that we publish. This form provides structure for consistency and transparency in reporting. For further information on Nature Portfolio policies, see our Editorial Policies and the Editorial Policy Checklist.

Statistics

For all statistical analyses, confirm that the following items are present in the figure legend, table legend, main text, or Methods section.

n/a	Confirmed	
☐	☒	The exact sample size (n) for each experimental group/condition, given as a discrete number and unit of measurement
☐	☒	A statement on whether measurements were taken from distinct samples or whether the same sample was measured repeatedly
☐	☒	The statistical test(s) used AND whether they are one- or two-sided<br>*Only common tests should be described solely by name; describe more complex techniques in the Methods section.*
☒	☐	A description of all covariates tested
☐	☒	A description of any assumptions or corrections, such as tests of normality and adjustment for multiple comparisons
☐	☒	A full description of the statistical parameters including central tendency (e.g. means) or other basic estimates (e.g. regression coefficient) AND variation (e.g. standard deviation) or associated estimates of uncertainty (e.g. confidence intervals)
☐	☒	For null hypothesis testing, the test statistic (e.g. F, t, r) with confidence intervals, effect sizes, degrees of freedom and P value noted<br>*Give P values as exact values whenever suitable.*
☒	☐	For Bayesian analysis, information on the choice of priors and Markov chain Monte Carlo settings
☒	☐	For hierarchical and complex designs, identification of the appropriate level for tests and full reporting of outcomes
☒	☐	Estimates of effect sizes (e.g. Cohen's d, Pearson's r), indicating how they were calculated

Our web collection on statistics for biologists contains articles on many of the points above.

Software and code

Policy information about availability of computer code

Data collection	Detailed information about cell culture and treatment, library preparation, and next generation sequencing are described in the materials and method section. Briefly, FACS Aria IIIu, FACS Canto II (BD), Bio-Rad CFX real-time PCR system, SMART-Seq v4 Ultra Low Input RNA Kit, NEBNext® Ultra™ DNA Library Prep Kit, Nextera DNA Sample Preparation kit, Nextseq 500 (Illumina) were used.
Data analysis	Data was analyzed using R version 4.0.4 with the following packages: DESeq2 (differential expression and accessibility analysis), Macs2 (version 2.2.6), limma-voom. Other tools used: FastQC v0.11.8, Bowtie2 v2.3.4.3, SAMtools v1.9, BEDtools v2.27.1, HOMER v4.11

For manuscripts utilizing custom algorithms or software that are central to the research but not yet described in published literature, software must be made available to editors and reviewers. We strongly encourage code deposition in a community repository (e.g. GitHub). See the Nature Portfolio guidelines for submitting code & software for further information.

Data

Policy information about availability of data

All manuscripts must include a data availability statement. This statement should provide the following information, where applicable:

- Accession codes, unique identifiers, or web links for publicly available datasets
- A description of any restrictions on data availability
- For clinical datasets or third party data, please ensure that the statement adheres to our policy

The NGS data generated in this study were deposited in the NCBI Gene Expression Omnibus as a SuperSeries under accession code GSE247722 [https://

www.ncbi.nlm.nih.gov/geo/query/acc.cgi?acc=GSE247722] composed of the following SubSeries: GSE247717 (iPS-macrophage RNA-seq), GSE247721 (RTE enrichment analysis of iPS-macrophages and PB-macrophages), GSE247710 (iPS-VEC RNA-seq), GSE247718 (iPS-VSMC RNA-seq), GSE247716 (ATAC-seq), and GSE247705 (CUT&TAG ChIP-seq). Source data are provided in this paper. The authors declare that all data supporting the findings of this study are available within the article and its Supplementary Information file.

Research involving human participants, their data, or biological material

Policy information about studies with human participants or human data. See also policy information about sex, gender (identity/presentation), and sexual orientation and race, ethnicity and racism.

Reporting on sex and gender	Not applicable. No sex- and gender-based analyses were performed in this study.
Reporting on race, ethnicity, or other socially relevant groupings	Not applicable.
Population characteristics	Not applicable.
Recruitment	Not applicable.
Ethics oversight	The ethics committee of Chiba University, Chiba, Japan (#1145) approved this study.

Note that full information on the approval of the study protocol must also be provided in the manuscript.

Field-specific reporting

Please select the one below that is the best fit for your research. If you are not sure, read the appropriate sections before making your selection.

☒ Life sciences ☐ Behavioural & social sciences ☐ Ecological, evolutionary & environmental sciences

For a reference copy of the document with all sections, see nature.com/documents/nr-reporting-summary-flat.pdf

Life sciences study design

All studies must disclose on these points even when the disclosure is negative.

Sample size	No statistical method was used to predetermine sample size. In this study all the experiments were performed using three (for healthy and gene-corrected (gc) Werner Syndrome (WS)) and four (for WS) individual iPSC clones obtained from three different age-matched healthy donors, WS patients, and their corresponding gene-corrected iPSCs.
Data exclusions	No data were excluded from the analyses.
Replication	All the experiments were performed with three (for healthy and gene-corrected (gc) Werner Syndrome (WS))or four (for WS) biologically independent samples from three different age-matched healthy donors, WS patients, and their corresponding gene-corrected iPSCs. As for the mRNA expression data, we confirmed the respective protein expression either by ELISA or immunocytochemistry, and all the results were consistent. In addition, the results of RNA-seq are were also corresponded with ATAC-seq. Moreover, we also validated the retrotransposable element expression in healthy, WS, and gcWS iPS-derived macrophages with healthy aged donor- and Werner syndrome patient-derived primary macrophages, and found all the results consistent.
Randomization	All the samples were randomly allocated into experimental groups. There is no bias when performing each experiment and collecting data.
Blinding	The investigators were not blinded since analyses relied on unbiased measurements of quantitative parameters. However, standardized procedures for data collection and analysis were used to prevent bias.

Reporting for specific materials, systems and methods

We require information from authors about some types of materials, experimental systems and methods used in many studies. Here, indicate whether each material, system or method listed is relevant to your study. If you are not sure if a list item applies to your research, read the appropriate section before selecting a response.

Materials & experimental systems

n/a	Involved in the study
☐	☒ Antibodies
☐	☒ Eukaryotic cell lines
☒	☐ Palaeontology and archaeology
☒	☐ Animals and other organisms
☒	☐ Clinical data
☒	☐ Dual use research of concern
☒	☐ Plants

Methods

n/a	Involved in the study
☐	☒ ChIP-seq
☐	☒ Flow cytometry
☒	☐ MRI-based neuroimaging

Antibodies

Antibodies used

APC anti-human CD34 (cat. #343508, Biolegend) (Clone: 561) (1:100 dilution)
APC/Cyanine7 anti-human CD34 (cat. #343514, Biolegend) (Clone: 581) (1:100 dilution)
APC anti-human CD43 (cat. #343206, Biolegend) (Clone: CD43-10G7) (1:100 dilution)
PE/Cyanine7 anti-human CD309 (VEGFR2) (cat. #359912, Biolegend) (Clone: 7D4-6) (1:100 dilution)
FITC anti-human TRA 1-60 (cat. #560173, BD Pharmingen) (Clone: 561) (1:50 dilution)
PE anti-human CD33 (cat. #303404, Biolegend) (Clone: WM53) (1:100 dilution)
PE/Cyanine7 anti-human CD14 (cat. #A22331, Backman Counter) (Clone: RMO52)(1:100 dilution)
APC anti-human CD16 (cat. #302023, Biolegend) (Clone: B73.1) (1:100 dilution)
FITC anti-human CD11b (cat. #301318, Biolegend) (Clone: ICRF44) (1:100 dilution)
APC anti-human HLA-DR (cat. #559866, BDBiosciences) (Clone: G46-6) (1:100 dilution)
PE anti-human CD163 (cat. #333606, Biolegend) (Clone: GHI/61) (1:100 dilution)
PE anti-human CD31 (cat. #303106, Biolegend) (Clone: WM59) (1:100 dilution)
APC anti-human CD144 (VE-cad) (cat. #348508, Biolegend) (Clone: BV9) (1:100 dilution)
Anti-alpha smooth muscle Actin (cat. #ab7817, Abcam) (Clone: 1A4) (1:1000 dilution)
Anti-Calponin-1 (cat. #abt129, Merck-Millipore) (1:500 dilution)
Anti-VE cadherin (cat. #ab33168, Abcam) (1:500 dilution)
Goat Anti-Mouse IgG H&L (Alexa Fluor® 488) (cat. #ab150113, Abcam) (1:200 dilution)
Goat Anti-Rabbit IgG H&L (Alexa Fluor® 555) (cat. #ab150078, Abcam) (1:200 dilution)
Phospho-Histone H2A.X (Ser139) (20E3) Rabbit mAb (cat. #9718, Cell Signaling) (1:200 dilution)
Histone H3K9me3 antibody (mAb) (cat. #ab150078, Abcam) (Clone: MABI 0319) (1:50 dilution)
Anti-dsRNA monoclonal antibody J2 (cat. #RNT-SCI-10010200, Jena Bioscience) (1:40 dilutions)
MAVS monoclonal antibody conjugated with APC (cat. #17-9835-41, eBioscience) (clone: ABM28H9) (1:50 dilutions)

Validation

All antibodies are validated by company. Validations are noted in technical data sheet of product pages, listed as follows:

https://www.biolegend.com/ja-jp/products/apc-anti-human-cd34-antibody-6204
https://www.biolegend.com/ja-jp/products/apc-cyanine7-anti-human-cd34-antibody-6159
https://www.biolegend.com/ja-jp/products/apc-anti-human-cd43-antibody-6246
https://www.biolegend.com/ja-jp/products/pe-cyanine7-anti-human-cd309-vegfr2-antibody-9748
https://www.bdbiosciences.com/en-us/products/reagents/flow-cytometry-reagents/research-reagents/single-color-antibodies-ruo/fitc-mouse-anti-human-tra-1-60-antigen.560380
https://www.biolegend.com/nl-be/products/pe-anti-human-cd33-antibody-878
https://www.beckman.jp/reagents/coulter-flow-cytometry/antibodies-and-kits/single-color-antibodies/cd14/a22331
https://www.biolegend.com/ja-jp/products/apc-anti-human-cd16-antibody-9053
https://www.biolegend.com/ja-jp/products/fitc-anti-human-cd11b-antibody-8299
https://www.bdbiosciences.com/ja-jp/products/reagents/flow-cytometry-reagents/research-reagents/single-color-antibodies-ruo/apc-mouse-anti-human-hla-dr.559866
https://www.biolegend.com/ja-jp/search-results/pe-anti-human-cd163-antibody-4793?GroupID=BLG9966
https://www.biolegend.com/ja-jp/search-results/pe-anti-human-cd31-antibody-882?GroupID=BLG5721
https://www.biolegend.com/en-us/search-results/apc-anti-human-cd144-ve-cadherin-antibody-6876
https://www.abcam.com/alpha-smooth-muscle-actin-antibody-1a4-ab7817.html
https://www.merckmillipore.com/JP/ja/product/Anti-Calponin-1-Antibody,MM_NF-ABT129
https://www.abcam.com/ve-cadherin-antibody-intercellular-junction-marker-ab33168.html
https://www.abcam.com/goat-mouse-igg-hl-alexa-fluor-488-ab150113.html
https://www.abcam.com/goat-rabbit-igg-hl-alexa-fluor-555-ab150078.html
https://www.cellsignal.com/products/primary-antibodies/phospho-histone-h2a-x-ser139-20e3-rabbit-mab/9718
https://www.activemotif.com/catalog/details/61013
https://www.jenabioscience.com/rna-technologies/rna-analysis-detection/dsrna-detection/rnt-sci-10010-anti-dsrna-monoclonal-j2
https://www.thermofisher.com/antibody/product/MAVS-Antibody-clone-ABM28H9-Monoclonal/14-9835-82

Eukaryotic cell lines

Policy information about cell lines and Sex and Gender in Research

Cell line source(s)

HEK293T

Authentication

The HEK293T was obtained from the Center for iPS Cell Research and Application (CiRA), Kyoto University, Kyoto, Japan. The

Authentication | use of cells was approved by the ethics committees at Chiba University.

Mycoplasma contamination | Tested negative for mycoplasma contamination.

Commonly misidentified lines (See ICLAC register) | The cell line used in this study is not in the registry of commonly misidentified lines.

Plants

Seed stocks | Not applicable.

Novel plant genotypes | Not applicable.

Authentication | Not applicable.

ChIP-seq

Data deposition

☒ Confirm that both raw and final processed data have been deposited in a public database such as GEO.

☒ Confirm that you have deposited or provided access to graph files (e.g. BED files) for the called peaks.

Data access links
May remain private before publication.
https://www.ncbi.nlm.nih.gov/geo/query/acc.cgi?acc=GSE247705

Files in database submission

iMac_NB2_4_ChIP_60bp.fastq.gz
iMac_NB3_2_ChIP_60bp.fastq.gz
iMac_NB5_2_ChIP_60bp.fastq.gz
iMac_WB1_5_ChIP_60bp.fastq.gz
iMac_WB1_10_ChIP_60bp.fastq.gz
iMac_WB3_1_ChIP_60bp.fastq.gz
iMac_WB1_5_182_KI_ChIP_60bp.fastq.gz
iMac_WB1_5_202_KI_ChIP_60bp.fastq.gz
iMac_WB1_10_KI_ChIP_60bp.fastq.gz
iMac_NB2_4_ChIP_60bp_d.9173_w200s100.bw
iMac_NB3_2_ChIP_60bp_d.7235_w200s100.bw
iMac_NB5_2_ChIP_60bp_d.6611_w200s100.bw
iMac_WB1_5_ChIP_60bp_d_w200s100.bw
iMac_WB1_10_ChIP_60bp_d.7398_w200s100.bw
iMac_WB3_1_ChIP_60bp_d.6765_w200s100.bw
iMac_WB1_5_182_KI_ChIP_60bp_d.6753_w200s100.bw
iMac_WB1_5_202_KI_ChIP_60bp_d.9988_w200s100.bw
iMac_WB1_10_KI_ChIP_60bp_d.6881_w200s100.bw

Genome browser session (e.g. UCSC) | UCSC hg19

Methodology

Replicates | Biologically independent samples

Sequencing depth | single-end

Antibodies | Histone H3K9me3 antibody (mAb) (cat. #ab150078, Abcam)

Peak calling parameters | Sequences were aligned to human genome sequences (hg19) using Bowtie2 (default setting).

Data quality | Duplicate, unmapped, or poor-quality reads, mitochondrial reads, and overlaps with the ENCODE blacklist were removed. Mapped reads were subsampled using samtools to make the numbers of reads in all samples the same.

Software | Data was analyzed using R version 4.0.4 with the following packages: DESeq2, Other tools used: Bowtie2 v2.3.4.3, SAMtools v1.9, BEDtools v2.27.1.

Flow Cytometry

Plots

Confirm that:

☒ The axis labels state the marker and fluorochrome used (e.g. CD4-FITC).

☒ The axis scales are clearly visible. Include numbers along axes only for bottom left plot of group (a 'group' is an analysis of identical markers).

☐ All plots are contour plots with outliers or pseudocolor plots.

☒ A numerical value for number of cells or percentage (with statistics) is provided.

Methodology

Sample preparation	On day 20 of differentiation, iPS-derived macrophages were serum-starved for 24-36 h and incubated for 6 h with DiI-oxLDL at a concentration of 50 µg/mL. As for the adherent cells, vascular endothelial cells and vascular smooth muscle cells, they were trypsinized to get single cells. Cells were then washed three times with PBS, resuspended in staining medium (PBS supplemented with 2% FBS), incubated for 30 min with appropriate antibodies on ice in the dark, washed with cold staining medium. Cells were then resuspended in staining medium with propidium iodide (PI), followed by sorting or analyze.
Instrument	FACS Aria IIIu, FACS Canto II (BD)
Software	FACS Diva application were used for collecting FACS Data. Data was then analyzed using FlowJo software v10
Cell population abundance	Cell sorting efficiency was confirmed by flow cytometric analysis of post-sorted cells.
Gating strategy	FCS-A/SSC-A were used for cell gating. Live cells were gated by propidium iodide (PI) negative.

☒ Tick this box to confirm that a figure exemplifying the gating strategy is provided in the Supplementary Information.
